# Seurat Spleen: A Pathognomonic Sign in Angiography

**DOI:** 10.7759/cureus.19439

**Published:** 2021-11-10

**Authors:** Jack B Newcomer, Gaby E Gabriel, Driss Raissi

**Affiliations:** 1 Interventional Radiology, University of Kentucky College of Medicine, Lexington, USA; 2 Radiology, University of Kentucky College of Medicine, Lexington, USA

**Keywords:** splenic artery embolization, splenic trauma, splenic infarcts, pseudoaneurysm of splenic artery, abdominal trauma, splenic injury, seurat spleen

## Abstract

Splenic injury commonly occurs following abdominal trauma and can result in severe complications and death if it goes unrecognized. The Seurat spleen is a term used to describe the angiographic appearance of splenic injury following blunt trauma, given its resemblance to the pointillistic artwork of French neo-impressionist Georges Seurat. We present a case of a 43-year-old man who presented following a motor vehicle collision and was found to have multiple punctate foci of contrast extravasation in the spleen consistent with the Seurat spleen angiographic sign. This angiographic pattern can be used as a pathognomonic sign to identify splenic injury, with early identification crucial to preventing further complications of the injury.

## Introduction

Splenic injuries are some of the most common injuries secondary to abdominal trauma [1,2]. The spleen is especially vulnerable due to its position behind the 9th, 10th, and 11th ribs in the left upper abdomen. Traumatic injuries can occur via penetrating trauma (such as gunshot wounds), blunt trauma, and indirect trauma, with the most common cause being motor vehicle collision (MVC). As the spleen is the most vascular organ of the body, significant hemoperitoneum can result from splenic injury, leading to death if it goes unrecognized. Delayed splenic rupture can be a fatal complication of splenic injury, occurring up to 10 days following the initial injury and often related to the subtle injury that is initially occult on imaging. Other complications include re-admission for bleeding, splenic artery pseudoaneurysm, and splenic abscess.

The Seurat spleen is a term coined by Kass and Fisher to describe the angiographic appearance of splenic injury following blunt trauma, given its resemblance to the pointillistic artwork of French neo-impressionist Georges Seurat (1859-1891) [3]. Pointillism represents a technique of painting where small and distinct dots of color are applied in patterns to form an image. The angiographic finding of innumerable punctate foci of contrast extravasation classically seen in splenic injury is analogous to Seurat’s technique of using a multitude of tiny dots that merge to form a clear singular image [4]. We present a case of a 43-year-old man who presented following an MVC and was found to have multiple punctate foci of contrast extravasation in the spleen consistent with the Seurat spleen angiographic sign.

## Case presentation

**Figure 1:**
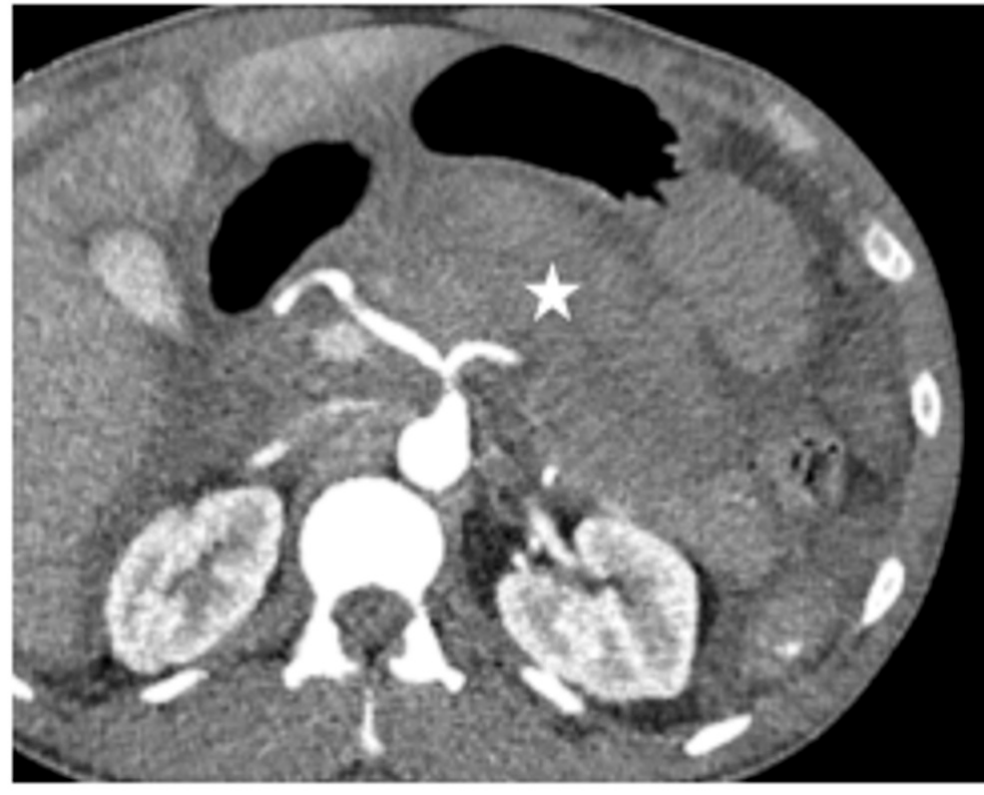
CT abdomen demonstrating a fluid collection in the lesser sac representing hemorrhage

A 43-year-old-male with no known medical problems presented from an outside hospital with concern for intraabdominal hemorrhage. He was in an MVC three weeks prior and presented with complaints of right upper quadrant abdominal pain, left-sided chest pain, nausea, and vomiting. A CT scan showed fluid in the lesser sac, suggestive of pancreatic hemorrhage, so he was transferred to the University of Kentucky Medical Center for further management (Figure 1).

**Figure 2:**
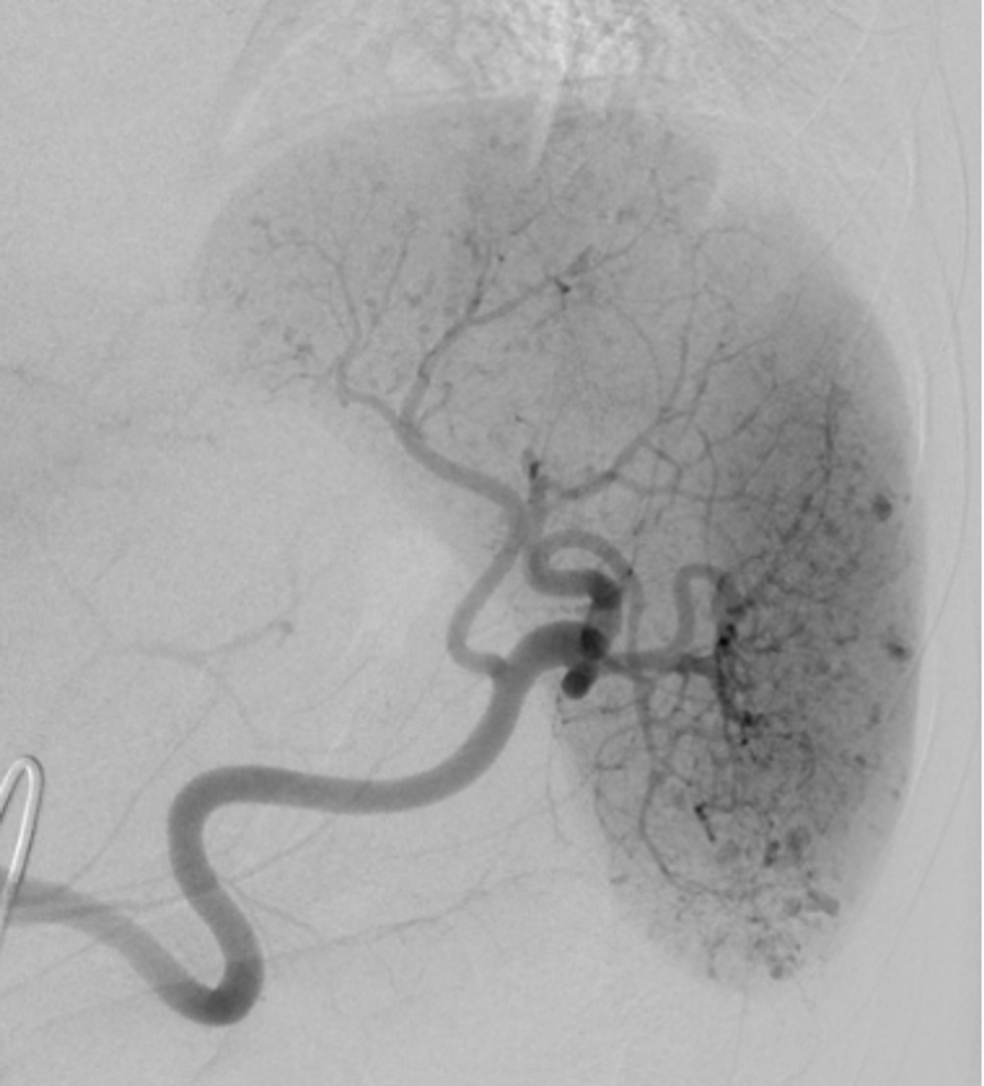
Celiac artery angiography revealing punctate foci of contrast extravasation in the splenic parenchyma consistent with multifocal tiny pseudoaneurysms

**Figure 3:**
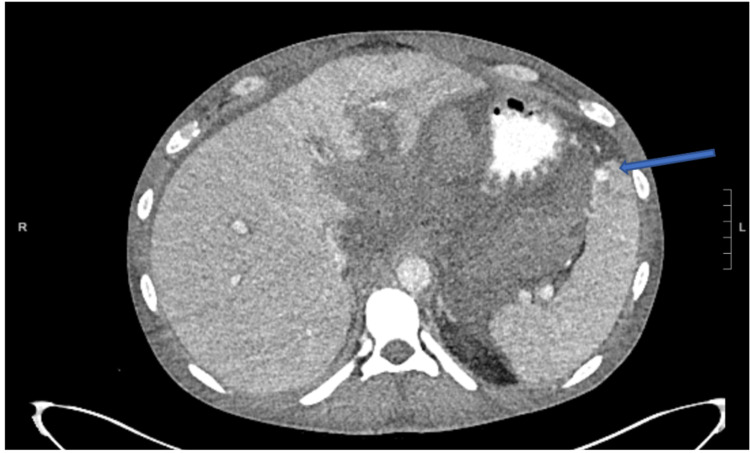
Contrast-enhanced axial CT of the abdomen demonstrating a focus of contrast opacification representing a pseudoaneurysm

Interventional radiology was consulted, and celiac angiogram showed an arterio-portal fistula in the liver which was embolized with 900 um particles Embozene (Palo Alto, CA: Varian Medical Systems, Inc.), as well as a few tiny rounded foci of contrast enhancement in the spleen which was managed conservatively without embolization (Figure 2). Following the procedure, the patient developed an elevated white blood cell (WBC) count and increased abdominal distension. A repeat CT scan four days later showed an increase in the size of the lesser sac hematoma and a pseudoaneurysm in the anterior spleen (Figure 3). Surgery was discussed with the patient, which he elected against and preferred to leave the hospital.

**Figure 4:**
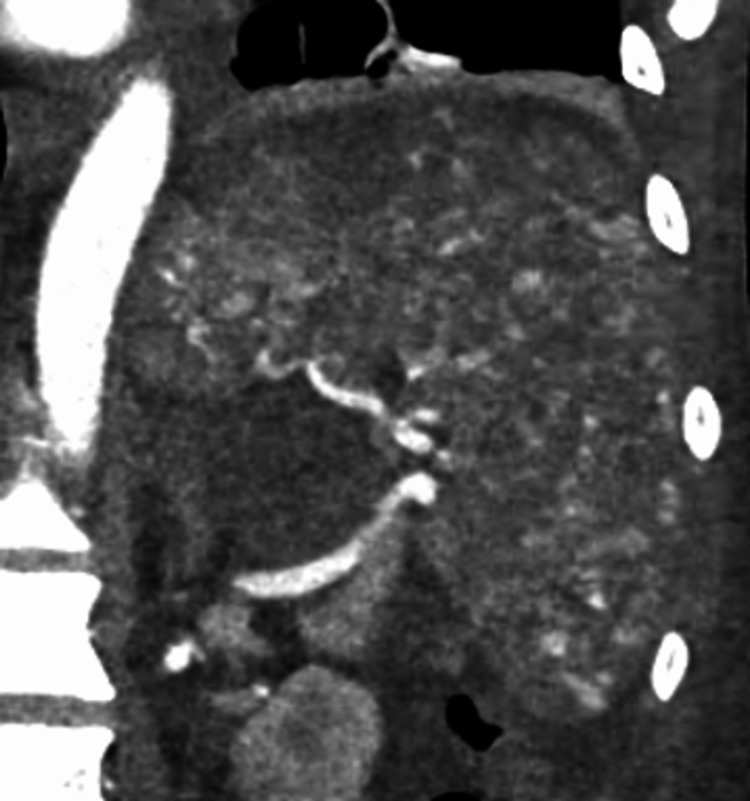
Innumerable small punctate foci of intraparenchymal contrast enhancement in a traumatic spleen on coronal CT

**Figure 5:**
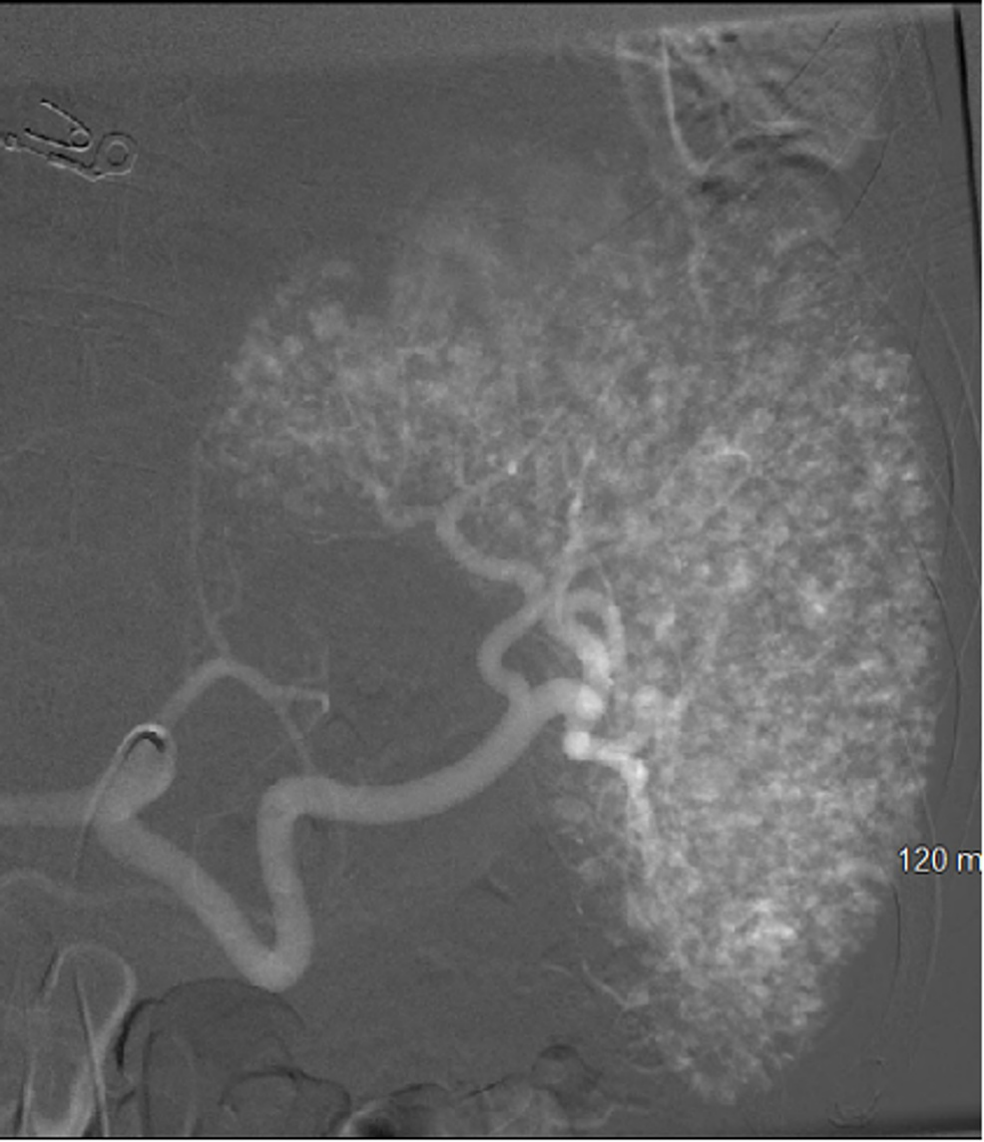
Innumerable small punctate foci of intraparenchymal contrast enhancement in a traumatic spleen on angiogram

He returned to the hospital two weeks later with decreased appetite, melena, hematochezia, abdominal pain, and weakness. He was found to be anemic with a hemoglobin of 6.4 g/dL (down from 7.8 g/dL). CT scan and angiographic images revealed worsening diffuse multifocal parenchymal perfusion abnormalities and innumerable punctate foci of contrast pooling in the spleen, compatible with high-grade splenic injury and the Seurat spleen angiographic sign (Figures 4, 5).

**Figure 6:**
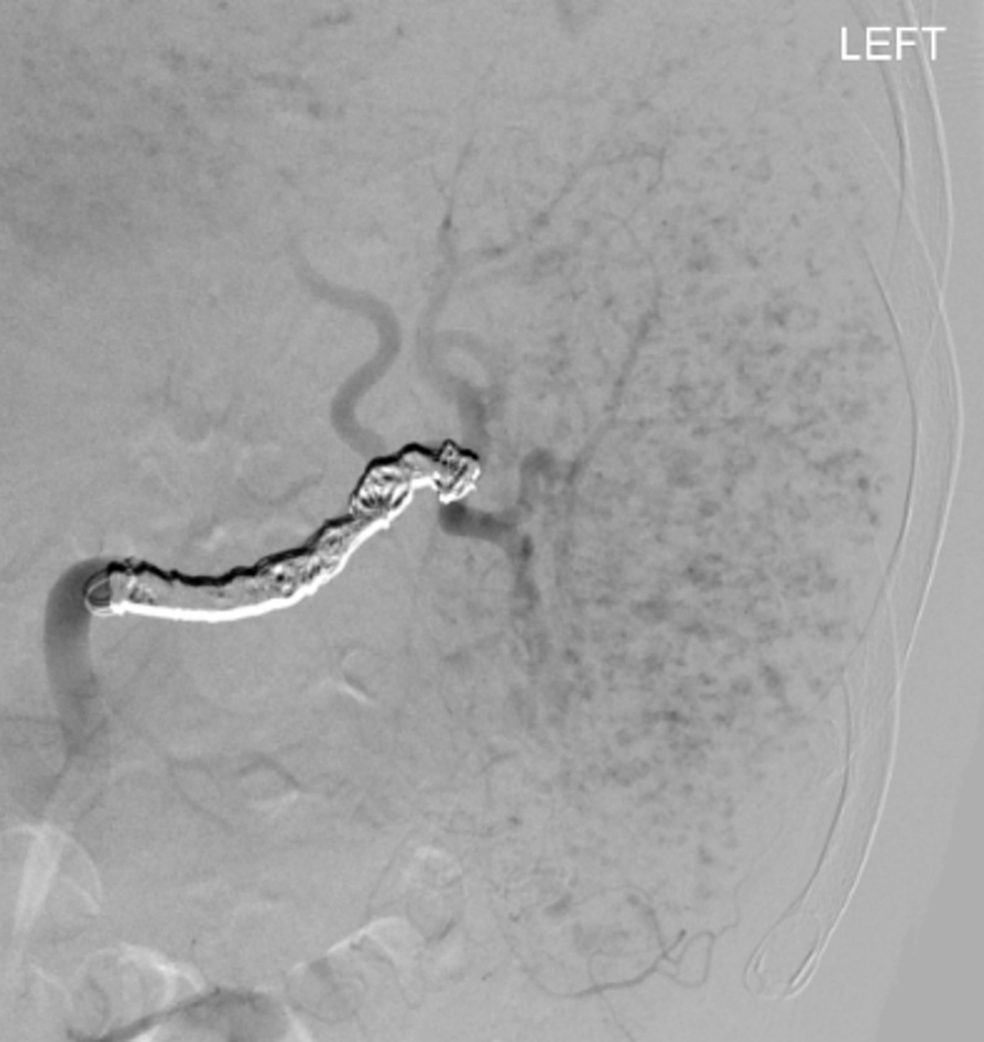
Postembolization angiogram showing decreased opacification of punctate foci of contrast extravasation

**Figure 7:**
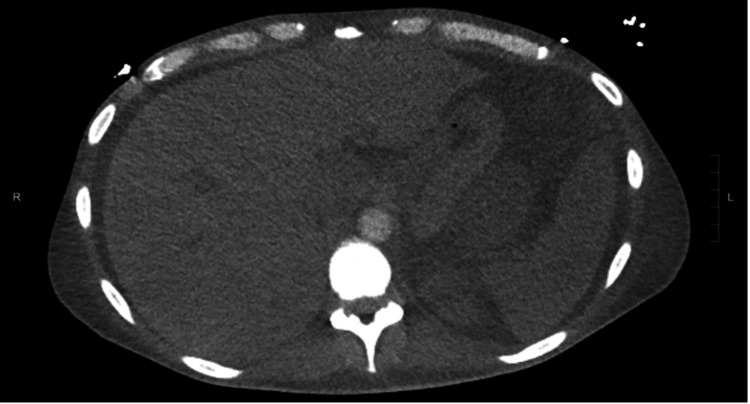
CT scan one month following splenic artery embolization showing no opacification of pseudoaneurysms within the splenic parenchyma

Coil embolization of the splenic artery was performed with postembolization angiography showing no anterograde flow through the splenic artery, significantly decreased opacification of the previously noted intraparenchymal pseudoaneurysms, and opacification of the spleen via collaterals (Figure 6). CT scan one month later revealed normal splenic parenchyma, with a persistent hematoma anterior and superior to the spleen (Figure 7).

The patient continued to have a complicated hospital course and was found to have multiple myeloma confirmed with a bone marrow biopsy specimen. He, unfortunately, passed away just over a month later due to complications from multiple myeloma.

## Discussion

Splenic injury commonly results from abdominal trauma, most frequently from motor vehicle accidents. The incidence of splenic injury in multiple myeloma is very rare, with only four cases of splenic rupture having been reported in the literature [5,6]. Splenomegaly can be a manifestation of multiple myeloma as well, but this often manifests with thrombocytopenia which was not present in our patient [7].

**Figure 8:**
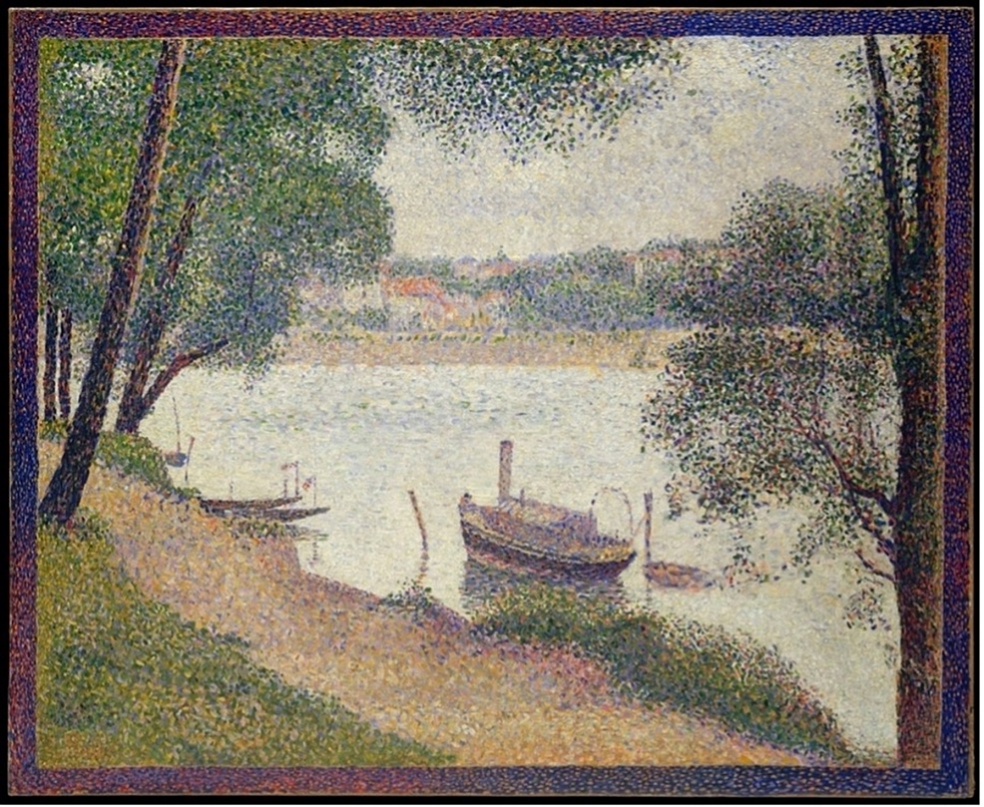
Painting representation of Seurat’s technique of pointillism showing a multitude of tiny dots merging together to form a clear singular image Seurat, Georges. Gray Weather, Grande Jatte. 1886-1888. The Walter H. and Leonore Annenberg Collection. The Metropolitan Museum of Art, New York

Splenic injury classically presents on angiography as innumerable punctate foci of contrast enhancement with or without extravasation. The pattern, which was present in our patient, is markedly similar to the works of Georges Seurat and his technique of pointillism, which consists of a multitude of tiny dots merging together to form a clear singular image (Figure 8). It is a very reliable sign of splenic injury [3]. Several mechanisms are thought to account for this mottled appearance, some of which include sinusoidal stasis, traumatic arteriovenous aneurysm, and contrast leakage from fragmented sinusoids [4,8]. Additional reliable indications of splenic injury include displacement and compression of splenic vessels and parenchyma by a hematoma, perfusion defects adjacent to parenchymal hyperemia indicating laceration, and early venous filling [9].

Splenic pseudoaneurysms can be a delayed complication of blunt splenic injury. In one review of 104 patients with splenic injury, delayed formation of splenic pseudoaneurysms occurred in 15.4% of patients and was detected on imaging between one to eight days following admission [10]. Our patient’s splenic pseudoaneurysms were detected on CT scan five days after his first admission, consistent with this previously observed range. These pseudoaneurysms were not present on a CT scan which was performed six days after the initial trauma in the emergency department. Splenic pseudoaneurysms can cause delayed splenic rupture and may support decisions to re-image following initial nonoperative management of splenic injury [11,12]. Splenic pseudoaneurysms herald a risk of rupture as high as 37% and mortality rates approaching 90% when untreated [13,14]. Early recognition of the signs and symptoms of delayed splenic rupture is essential to achieve the best possible outcomes for blunt splenic trauma patients.

Splenic artery embolization is a well-validated technique in the nonoperative management of hemodynamically stable patients presenting with blunt splenic trauma [11,15,16]. Nonoperative management is significantly more successful with adjunctive use of embolization, with higher rates of splenic salvage [17] and lower rates of hospital mortality and mean hospital stay [18]. Additional advantages of embolization compared to operative management include not only the elimination of surgical risks and complications but also the additional benefit of the preservation of splenic tissue. Hemodynamic instability is an absolute contraindication to nonoperative management. Surgical management is also recommended for patients with higher-grade injuries, patients older than 55 years, and patients who cannot be adequately observed or are unlikely to tolerate a significant episode of hypotension. Although our patient, unfortunately, passed away due to complications from multiple myeloma, the splenic artery embolization procedure was successful with normal splenic parenchyma noted on follow-up CT one month after.

## Conclusions

In summary, diffuse splenic vascular injury is common following blunt abdominal trauma and can lead to preventable death. The Seurat spleen is a reliable angiographic depiction of diffuse splenic injury and leaves little diagnostic doubt in the correct clinical setting. The angiographic image of diffuse small punctate foci of intraparenchymal contrast enhancement is akin to Seurat’s painting technique of pointillism, and this pattern can be used as a pathognomonic sign to identify splenic vascular injury.

## References

[1] Zarzaur BL, Rozycki GS (2017). An update on nonoperative management of the spleen in adults. Trauma Surg Acute Care Open.

[2] Yang K, Li Y, Wang C, Xiang B, Chen S, Ji Y (2017). Clinical features and outcomes of blunt splenic injury in children: a retrospective study in a single institution in China. Medicine (Baltimore).

[3] Kass JB, Fisher RG (1979). The Seurat spleen. AJR Am J Roentgenol.

[4] Scatliff JH, Fisher ON, Guilford WB, McLendon WW (1975). The "starry night" splenic angiogram. Contrast material opacification of the malpighian body marginal sinus circulation in spleen trauma. Am J Roentgenol Radium Ther Nucl Med.

[5] Mello DF, Costa MJ, Febronio EM, Landell GA, Queiroz RM (2020). Spontaneous splenic rupture associated with multiple myeloma: a serious and unusual case. Hematol Transfus Cell Ther.

[6] Hatzimichael E, Benetatos L, Stebbing J, Kapsali E, Panayiotopoulou S, Bourantas KL (2006). Spontaneous splenic haematoma in a multiple myeloma patient receiving pegfilgrastim support. Clin Lab Haematol.

[7] Ryzhko VV, Klodzinskiĭ AA, Grachev AE, Varlamova EIu, Sataeva MS (2013). Multiple myeloma predominantly involving the spleen. [Article in Russian]. Ter Arkh.

[8] Brindle MJ (1972). Arteriography and minor splenic injury. Clin Radiol.

[9] Gold RE, Redman HC (1972). Splenic trauma: assessment of problems in diagnosis. Am J Roentgenol Radium Ther Nucl Med.

[10] Muroya T, Ogura H, Shimizu K (2013). Delayed formation of splenic pseudoaneurysm following nonoperative management in blunt splenic injury: multi-institutional study in Osaka, Japan. J Trauma Acute Care Surg.

[11] Kluger Y, Rabau M (1998). Improved success in nonoperative management of blunt splenic injuries: embolization of splenic artery pseudoaneurysm. J Trauma.

[12] Norotsky MC, Rogers FB, Shackford SR (1995). Delayed presentation of splenic artery pseudoaneurysms following blunt abdominal trauma: case reports. J Trauma.

[13] Huang IH, Zuckerman DA, Matthews JB (2004). Occlusion of a giant splenic artery pseudoaneurysm with percutaneous thrombin-collagen injection. J Vasc Surg.

[14] LiPuma JP, Sachs PB, Sands MJ, Stuhlmiller S, Herbener TE (1997). Angiography/interventional case of the day. Splenic artery pseudoaneurysm associated with pancreatitis. AJR Am J Roentgenol.

[15] Schurr MJ, Fabian TC, Gavant M (1995). Management of blunt splenic trauma: computed tomographic contrast blush predicts failure of nonoperative management. J Trauma.

[16] Becker CD, Spring P, Glättli A, Schweizer W (1994). Blunt splenic trauma in adults: can CT findings be used to determine the need for surgery?. AJR Am J Roentgenol.

[17] Banerjee A, Duane TM, Wilson SP (2013). Trauma center variation in splenic artery embolization and spleen salvage: a multicenter analysis. J Trauma Acute Care Surg.

[18] Rajani RR, Claridge JA, Yowler CJ (2006). Improved outcome of adult blunt splenic injury: a cohort analysis. Surgery.

